# Challenges of Implementing the PRISM 2.0 Clinical Trial With Assisted Living and Impaired Participants

**DOI:** 10.1093/geroni/igab046.1192

**Published:** 2021-12-17

**Authors:** Joseph Sharit, Sara Czaja, Jerad Moxley, Carlos Almirola

**Affiliations:** 1 Department of Industrial Engineering, University of Miami, Coral Gables, Florida, United States; 2 Weill Cornell Medicine/Center on Aging and Behavioral Research, New York, New York, United States; 3 Weill Cornell Medicine, New York, New York, United States; 4 University of Miami Miller School of Medicine, Miami, Florida, United States

## Abstract

The PRISM 2.0 clinical trial examined the benefits of a software system, implemented on a computer tablet, which was designed to support access to information, engagement, and social connectivity among older people. Participants across three sites were recruited from rural locations, senior living housing facilities, and assisted living facilities (ALFs) and correspondingly randomized into either the Prism or control (tablet computer without the PRISM system) conditions. In this talk, we focus on the challenges associated with including ALF participants at key stages of the trial. These stages included telephone prescreening, baseline assessment, training on the system, and 6-, 9-, and 12-month follow-up assessments. Inability to meet inclusion criteria related to cognitive and sensory-motor considerations was a common problem, as was the ability to sustain attention during the training sessions. Recommendations for recruitment and retaining older adults in ALFs for these types of studies will be offered.

